# MicroRNA regulator gene mutations in thyroid follicular nodular disease and thyroid cancer: does it all come down to timing?

**DOI:** 10.1530/ETJ-24-0298

**Published:** 2024-12-19

**Authors:** Vincenzo Condello, C Christofer Juhlin

**Affiliations:** ^1^ Department of Oncology-Pathology, Karolinska Institutet, Stockholm, Sweden; ^2^ Department of Pathology and Cancer Diagnostics, Karolinska University Hospital, Stockholm, Sweden

**Keywords:** *DICER1*, *DGCR8*, *AGO2*, *XPO5*, *DROSHA*, microRNA, thyroid tumor, mutation

## Abstract

In recent years, germline mutations in the microRNA (miRNA) processor genes *DICER1* and *DGCR8* have been coupled to the development of thyroid follicular nodular disease (TFND), thereby casting new light on the etiology of this enigmatic, benign condition in non-iodine-deficient regions. Moreover, *DICER1* and *DGCR8* mutations have also been reported in rare subsets of follicular cell-derived thyroid carcinomas. Specifically, truncating germline or missense somatic *DICER1* mutations have been reported in small subsets of pediatric and adolescent follicular thyroid carcinoma (FTC) and poorly differentiated thyroid carcinoma (PDTC). Similarly, a recurrent somatic mutation of the *DGCR8* gene has been observed in highly aggressive FTCs and in some indolent cases of encapsulated follicular variant of papillary thyroid carcinoma. The reason why identical mutations in the same miRNA processor gene can lead to such a myriad of thyroid conditions, ranging from benign TFND to FTCs and PDTCs, remains unclear. This review highlights key features of miRNA regulator gene mutations in thyroid disease and explores their potential roles as drivers or progression events in tumor development.

## MicroRNAs in human biology

MicroRNAs (miRNAs) are a highly conserved class of endogenous small noncoding RNAs (snRNAs), approximately 22 nucleotides in length, that function as negative regulators of protein-coding gene expression. Each miRNA can regulate hundreds of mRNA targets, although the typical regulation level ranges from 30 to 50% (1).

The regulatory effects of a small RNA were first proposed in 1993 by two independent groups studying *Caenorhabditis elegans* (2). These studies demonstrated that a tiny RNA, named lin-4, could act as a post-transcriptional regulator influencing the expression of the *lin-4* gene. Since then, compelling evidence further demonstrated and supported the importance of miRNAs in human biology, opening new lines of inquiry in this emerging field (3). In particular, seven years later, Reinhart and coworkers successfully showed that *let-7*, another *Caenorhabditis elegans* heterochronic gene, was also represented by an snRNA. Together with *lin-4*, *let-7* was able to trigger the cascade of regulatory heterochronic genes (4). This discovery spurred research groups to investigate other snRNAs.

Nowadays, the role of miRNAs in human biology and pathophysiology has been elucidated by specific studies, showing first that miRNAs may exhibit a tissue-specific expression pattern (5). miRNAs appear to be directly or indirectly involved in regulating a wide spectrum of biological functions, ranging from cell cycle, differentiation, proliferation, apoptosis, stress tolerance and energy metabolism to the immune response (5). Furthermore, a rapidly growing number of studies provided evidence that amplification or deletion of miRNA genes, abnormal transcriptional control of miRNAs, changes in epigenetic regulation and defects in the miRNA biogenesis machinery represent the principal mechanisms through which miRNA expression becomes dysregulated in tumors (6). Instances of global miRNA downregulation in tumors have been observed, and one of the first explanations proposed for this widespread underexpression in cancer cells is related to the role of many miRNAs in maintaining lineage-specific characteristics. Their reduced abundance is thought to promote a dedifferentiated state in tumor cells, thereby enhancing their aggressive potential (6, 7). Consequentially, the dysregulated miRNAs have been shown to influence the most important hallmarks of cancer, including proliferative signaling, evading growth suppressors, resisting apoptosis and activating aggressive behaviors, such as invasion, angiogenesis and metastasis (8).

In the early 2000s, Dr Croce’s group provided the first evidence of miRNA involvement in human cancer. Their studies focused mainly on the characterization of chromosome 13q14 deletion in human B-cell chronic lymphocytic leukemia, which represents the most common form of adult leukemia. It was observed that the two miRNAs *miR-15a* and *miR-16-1a* were the only two genes located on that small region of chromosome 13, commonly deleted in most B-cell chronic lymphocytic leukemia cases. Detailed expression analysis indicated that both miRNAs were absent or downregulated in ∼68% of patients affected by chronic lymphocytic leukemia, suggesting a potential role as tumor suppressors (9). Further studies revealed then that the two miRNAs *miR-15* and *miR-16-1* played an important role as tumor suppressors by repressing Bcl-2, a protein overexpressed in many solid tumors that acts as an anti-apoptotic regulator (10). In this regard, miRNAs may operate as tumor suppressors and oncogenes (oncomiRs) under specific conditions and according to different tissues and organs (9, 11).

## The biogenesis of miRNAs

The biogenesis of miRNAs is a multistep process involving multiple players. The evolution flows from the transcription of long double-stranded RNAs (dsRNAs) to the synthesis of the final functional regulators through several steps of maturation (1).

### The canonical pathway

**Figure 1:**
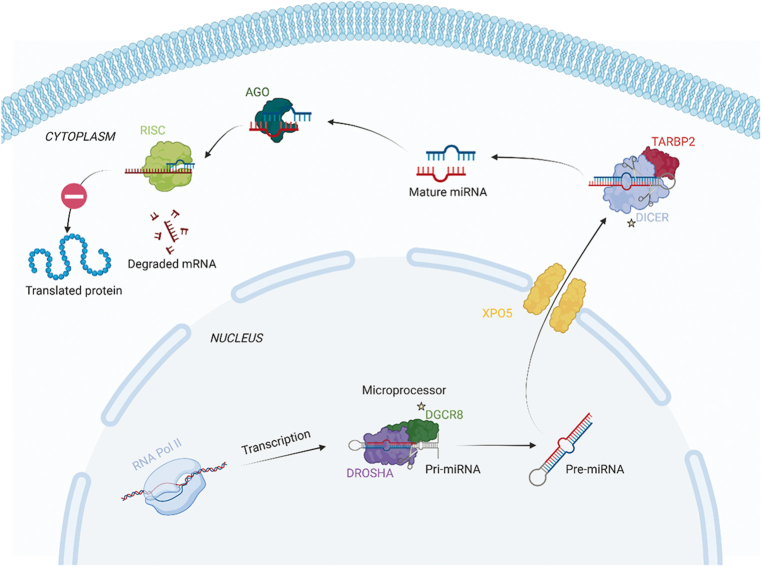
Canonical pathway illustration. Cellular regulation of microRNA (miRNA) maturation. Transcription of miRNA gene *via* RNA polymerase II (RNA Pol II) leads to the formation of a pri-miRNA molecule, which is then further transformed into pre-miRNA by the microprocessor complexes DGCR8 and DROSHA. The pre-miRNA is then exported to the cytosol *via* the nuclear membrane protein XPO5 and subsequently cleaved into mature miRNA by DICER1 and TARBP2. These molecules may inhibit specific mRNA molecules *via* RISC and AGO2. Genes known to be mutated in thyroid carcinoma are highlighted with stars. The figure was created with BioRender.com.

The canonical pathway is the leading biogenesis pathway by which miRNAs are processed (Fig. 1). Biogenesis begins in the nucleus, where miRNA gene transcription is mediated by RNA polymerase II (RNA Pol II), which synthesizes longer hairpin-like primary transcripts known as pri-miRNA. The hairpin-loop structure of pri-miRNAs is then recognized by a tandem of interacting proteins that together form the microprocessor complex, initiating the first step of miRNA maturation. The ribonuclease DROSHA, along with its cofactor DGCR8, cleaves the pri-miRNAs into small hairpin-structured precursors, known as pre-miRNAs, which are ∼70 nucleotides in length. Following this initial processing by the microprocessor complex, the pre-miRNAs are exported from the nucleus to the cytoplasm across the nuclear pores by the carrier protein exportin 5 (XPO5). In the cytoplasm, they undergo further processing by a second ribonuclease, DICER1, in conjunction with its cofactor TARBP2. This processing involves the removal of the terminal loop, resulting in dsRNA of approximately 22 nucleotides. These small dsRNAs are loaded onto specific Argonaute (AGO) proteins to assemble the RNA-induced silencing complex (RISC) loading complex (RLC). The final step of this process occurs when RLC binds to the 3′ UTR of the mRNA target, leading to gene silencing through translational repression or mRNA degradation (12).

### The noncanonical pathway

To date, several alternative miRNA biogenesis pathways, known as noncanonical pathways, have been described. These noncanonical pathways are generally categorized into two main types: microprocessor-independent and DICER1-independent pathways (13).

Mirtrons are a type of miRNA generated during the splicing of mRNA introns and do not require the activity of the DROSHA/DGCR8 complex. After being cleaved by the splicing machinery, the pre-miRNA is further processed and linearized by the enzyme DBR1, enabling the introns to adopt a structure that facilitates their transport to the cytoplasm by XPO5. Once in the cytoplasm, these mirtrons are recognized and cleaved by DICER1 to form a mature miRNA (12, 14).

In a DICER1-independent biogenesis pathway, miRNAs are processed by DROSHA. The resulting pre-miRNA has a stem-loop structure that is too short to be cleaved by DICER1. Instead, slicer activity of AGO2 is required to complete its maturation in the cytoplasm (15).

## Aberrant miRNA expression in thyroid diseases

Over the past decades, with the implementation of high-throughput technologies, the widespread dysregulation of miRNAs has emerged as a common feature of human tumors, taking center stage in molecular pathology and oncology. Specific subsets of dysregulated miRNAs have been identified across various human cancers, suggesting that aberrant miRNA expression may serve as a crucial hallmark of tumor development and progression (7). In this respect, thyroid tumors may serve as an intriguing model for investigation, considering the various grades of differentiation and the wide range of morphological and histopathological subtypes that predominantly originate from the same cell type (9).

Thyroid cancer has emerged as the most prevalent endocrine malignancy. In the recent edition of the WHO classification of endocrine and neuroendocrine tumors, follicular cell-derived thyroid tumors are categorized into three major groups, each comprising several subcategories. Follicular thyroid adenomas (FTAs) and their subtypes, together with the thyroid follicular nodular disease (TFND), were all included in the group of benign tumors; noninvasive follicular thyroid neoplasm with papillary-like nuclear features (NIFTP), tumors of uncertain malignant potential (WDT-UMP and FT-UMP) and hyalinizing trabecular tumor are all included in the low-risk neoplasms; and differentiated thyroid carcinomas (DTCs), such as papillary (PTC), oncocytic (OTC) and follicular (FTC) types, together with poorly (PDTC) and anaplastic (ATC) types are included in the group of malignant tumors (16).

Beyond the established role of constitutive activation of the MAPK and PI3K–AKT pathways, there exists a significant need to unveil and explore alternative mechanisms driving the evolution and progression of thyroid cancer. In recent years, substantial insights have been gained into the miRNA expression profiles in thyroid neoplasms. Given the increased role of miRNAs in determining cancer phenotype, the association between miRNA expression, molecular subtypes and clinical parameters has become a focal point of interest for many research groups (17, 18).

Numerous studies have assessed the potential diagnostic and prognostic role of miRNA expression in patients with benign, low-risk or malignant thyroid nodules, frequently comparing the mutational status and miRNA expression. In this context, in 2005, de la Chapelle and his research group provided the first evidence of specific miRNAs transcriptionally upregulated in PTCs compared with normal tissues. In particular, a set of five miRNAs, including the three most upregulated *miR-221*, *miR-222* and *miR-146*, unequivocally distinguished between tumors and normal thyroid (19). Soon thereafter, the deregulation of a specific group of miRNAs was identified in additional forms of DTC and dedifferentiated thyroid cancers (20, 21). A few years later, similar to mRNA profiles, Nikiforova and coworkers demonstrated how the various histopathological types of thyroid tumors may display distinct miRNA profiles, which further differ within the same tumor type driven by different oncogenic alterations, such as *BRAF*, *RAS*, *RET::PTC* and *PAX8::PPARG* (22).

In the past decade, Basolo’s group extensively examined the miRNA landscape in thyroid nodules. A miRNA signature that distinguishes the encapsulated form from the infiltrative form of follicular variant PTC (EFVPTC) was proposed (23). Soon thereafter, the same research group suggested that miRNA expression profiles of NIFTPs might vary based on their mutational status. Specifically, the miRNA expression profile of *BRAF*/*RAS* wild-type NIFTPs resembles that of FTAs, while NIFTPs carrying *RAS* or *BRAF* gene mutations exhibit a miRNA expression profile similar to that of infiltrative and invasive FVPTCs (24). Furthermore, a significant difference in miRNA expression profiles between nonmetastatic vs metastatic FVPTCs was observed (17). Analogously, a signature of dysregulated miRNAs was proposed in ATCs, suggesting that it might distinguish undifferentiated from poorly and well-differentiated carcinomas on the molecular level (25).

Moreover, The Cancer Genome Atlas (TCGA) research network further captured the complexity of this scenario, revealing the presence of six clusters in PTCs based on miRNA expression. *RAS*-like tumors were grouped in one cluster (cluster 1), whereas *BRAF*-like tumors were clustered separately in several clusters (clusters 2–6), and among these, tumors with a high risk of recurrence were grouped all together (clusters 5 and 6) (26). Of the miRNAs with cancer relevance, *miR-21*, *miR-146b* and *miR-204* were found to be highly correlated with the thyroid dedifferentiation score. *miR-21* was considered the most negatively correlated miRNA in both the entire and *BRAF*-like cohorts. Moreover, *miR-21* and *miR-146b* were already described as oncogenic miRNAs in several tumor types, whereas *miR-204* was described as downregulated in several tumor types with a potential role of tumor suppressor (7, 27).

The aberrant expression of miRNAs in thyroid diseases underscores their potential as prognostic markers, providing valuable insights into disease classification and progression. Moreover, this dysregulation opens up promising avenues for the development of targeted therapeutic interventions in thyroid tumors, potentially transforming clinical approaches and improving patient outcomes.

## DICER1 and familial multinodular goiter with schwannomatosis (FMGS) syndromes

**Figure 2:**
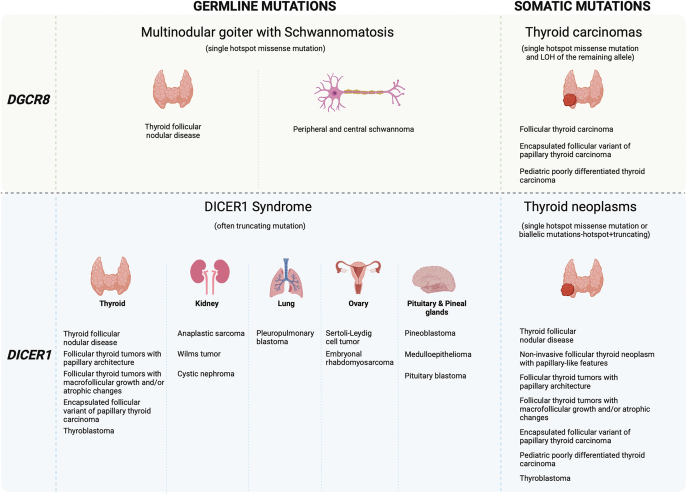
Proposed scheme for miRNA processor gene mutations and their functional consequences in thyroid follicular nodular disease and thyroid neoplasms. The figure was created with BioRender.com.

DICER1 syndrome is a multitumor syndrome characterized by a wide range of neoplastic and non-neoplastic conditions. The journey began with researchers discovering germline loss-of-function mutations in the RNase IIIb domain of the *DICER1* gene in families affected by pleuropulmonary blastoma (PPB), a rare childhood lung malignancy (28). However, PPB was just one aspect of this familial tumor predisposition condition. Subsequent investigations revealed both germline and somatic *DICER1* mutations in various other tumors associated with this syndrome, spanning multiple extrapulmonary sites. Examples of tumors apart from PPB that may arise in a DICER1 kindred include ovarian Sertoli–Leydig cell tumors, cystic nephromas, thyroid neoplasms and embryonal rhabdomyosarcoma, particularly affecting the cervix and the uterine corpus (Fig. 2). Moreover, DICER1 kindred often displayed nontumorous manifestations, such as macrocephaly and renal and retinal abnormalities. The estimated prevalence of DICER1 syndrome is roughly 1 in 11,000 in the general population, but approximately 1 in 5000 among cancer patients (29).

**Figure 3:**
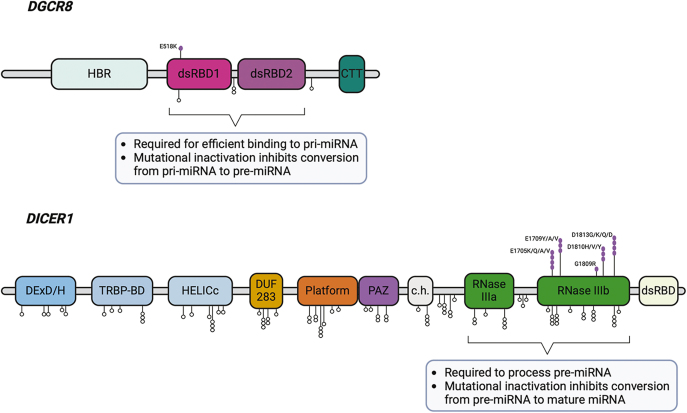
Schematic overview of the hotspot *DICER1* and *DGCR8* gene mutations in relation to functional domains. The figure was created with BioRender.com.

The DICER1 syndrome is an autosomal dominant condition, and most tumors arise in individuals who have inherited a *DICER1* loss-of-function (usually nonsense or frameshift) mutation and subsequently acquired a somatic missense mutation in one of the five hotspot codons within the RNase IIIb domain (E1705, D1709, G1809, D1810 and E1813) (Fig. 3). These mutations have been shown to lead to an imbalance in miRNA production, favoring the generation of 3p miRNA strands while reducing the production of 5p miRNA strands, but the downstream consequences on tumor formation are not clearly understood (18, 30, 31).

It seems likely that biallelic inactivation of both *DICER1* alleles is required for the tumor to develop properly, as this phenomenon has been reported for various DICER1-related neoplasia, such as PPBs, Wilms tumors, embryonal rhabdomyosarcoma and anaplastic sarcomas of the kidney, and in different subtypes of thyroid cancer (31, 32, 33, 34, 35, 36, 37).

The familial multinodular goiter with schwannomatosis (FMGS) syndrome is a recently described entity. The first report of this syndrome was published in 2020, in which the authors presented an index family with six individuals across three generations (38). All six patients developed TFND (described in the current article as ‘multinodular goiter’), and five had one or more peripheral nerve schwannomas (Fig. 2). In addition, one patient was diagnosed with choroid plexus papilloma at the age of seven. After ruling out *DICER1* mutations as the causative event, next-generation sequencing identified a shared germline variant (c.1552G>A, p.E518K) in *DGCR8* among all affected family members. *DGCR8* encodes a protein that functions as a miRNA processor, as described in detail in previous sections (Fig. 3). The variant caught the investigator’s attention because it is reported as a recurrent somatic mutation in Wilms tumors. Moreover, it is considered pathogenic by *in silico* analyses and is not found in the germline DNA of healthy individuals. Intriguingly, all somatic tissues obtained from the family (TFND, schwannomas and the single papilloma) exhibited biallelic inactivation of *DGCR8*, with loss of heterozygosity (LOH) of the remaining allele. This finding suggests a tumor-suppressive function for *DGCR8*. The estimated prevalence of FMGS is not known.

## Could *DICER1* mutations orchestrate the development of thyroid follicular nodular disease?

**Figure 4:**
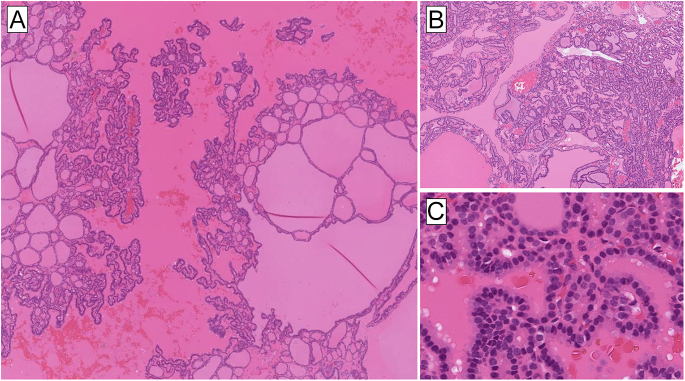
Thyroid follicular nodular disease (TFND) in a patient with a *DICER1* germline mutation presents with the classic histological features of TFND. However, DICER1-associated lesions may be distinguished by prominent papillary infoldings (A) and a striking heterogeneous mix between hyperplastic nodules (B) and classic colloid nodules. Notably, there is an absence of nuclear atypia (C).

Across various endocrine tumors, the identification of somatic driver gene mutations in sporadic tumors often stems from linking a specific gene mutation at the constitutional level to a syndrome where the particular endocrine tumor is overrepresented. For example, the identification of germline *MEN1* mutations in multiple endocrine neoplasia type 1 syndrome and *RET* mutations as causative agents of multiple endocrine neoplasia type 2 syndrome led to the subsequent discovery of somatic *MEN1* and *RET* mutations in sporadic parathyroid adenoma and medullary thyroid carcinoma, respectively, which are two main tumor manifestations of the respective syndromes. Similarly, somatic *NF1* and *VHL* mutations in pheochromocytoma were well identified after the initial observations of constitutional *NF1* and *VHL* gene mutations causing neurofibromatosis type 1 and von Hippel–Lindau syndromes. In a similar way, the identification of *DICER1* and *DGCR8* mutations underlying DICER1 and FMGS syndromes, respectively, paved the way for extended analyses of *DICER1* and *DGCR8* mutations in somatic tissues from patients with thyroid disease. TFND is one of the most common manifestations of DICER1 and FMGS syndromes, and this umbrella term incorporates several entities previously annotated as ‘multinodular goiter’ and ‘adenomatoid goiter’. Interestingly, a recent Danish study screening for *DICER1* variants in germline DNA from TFND patients <25 years showed that 13% of patients harbored pathogenic *DICER1* mutations (39), suggesting that *DICER1* genetic screening could be motivated in cases with early-onset TFND irrespective of family history. To build on this, the majority of patients with germline *DICER1* syndrome have been shown to harbor additional somatic *DICER1* mutations in hyperplasic areas within a TFND (40), suggestive of clonal events. *DICER1*-associated TFND may show prominent papillary infolding and multiple hyperplastic nodules, which could potentially be a morphological clue triaging patients for *DICER1* genetic screening (37) (Fig. 4).

This is not entirely surprising, as subsets of nodules within the spectrum of TFND have previously been shown to carry clonal mutations in genes such as *NRAS*, *HRAS*, TSH receptor (*TSHR*) and *GNAS* (41, 42). Thus, it is evident that germline *DICER1* mutations are tightly coupled to the development of TFND, with additional somatic mutations often observed in hyperplastic areas. The mechanism by which *DICER1* inactivation disrupts normal thyroid follicular architecture is not currently known. However, it is noteworthy that thyrocyte-specific *DICER1* knockout mice exhibit impaired follicular organization and increased fibrosis, suggesting a *DICER1*-dependent role in the development and maintenance of thyroid gland structure.

## *DICER1*-mutated follicular cell-derived thyroid carcinomas

Somatic and germline *DICER1* mutations in thyroid cancer are relatively rare but have been observed in specific contexts, often associated with distinct tumor subtypes and clinical parameters. Therefore, while the overall prevalence of *DICER1* alterations in thyroid neoplasia is very low, it is important to recognize that these mutations are enriched in pediatric and adolescent tumors with an aggressive clinical course and in follicular thyroid tumors exhibiting specific histological attributes, all reviewed below.

**Figure 5:**
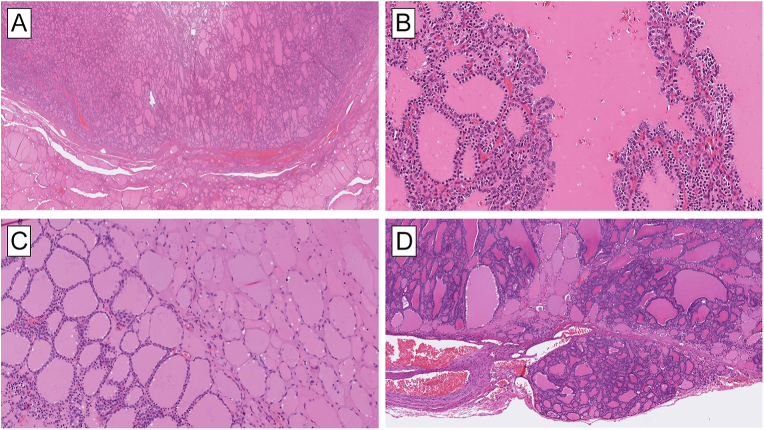
Histological harbingers of *DICER1* mutations in thyroid lesions. (A) Thyroid tumors with a predominant macrofollicular pattern are overrepresented in terms of *DICER1* mutations. Note the well-circumscribed lesion with a peripheral capsule. This was a macrofollicular thyroid adenoma with a single *DICER1* gene hotspot mutation. (B) Papillary structures are common findings in *DICER1*-aberrant thyroid lesions. (C) Abortive changes (right part of the image) as seen in this minimally invasive macrofollicular thyroid carcinoma are characterized by palely stained tumor cells with flat nuclei and surrounding hyaline-like stroma. This histological finding is strongly associated with *DICER1* mutational inactivation. Note the uninvolved tumor cells (left) shown for comparison. (D) Abortive changes may also be seen in *DICER1*-mutated FTC with normal follicular width.

Initially, DICER1 kindred with differentiated thyroid carcinoma were shown to carry additional somatic hotspot *DICER1* mutations, suggesting a biallelic inactivation pattern for thyroid tumors arising in a syndromic setting (36). Subsequent studies aiming to characterize *DICER1* gene status in sporadic tumors have described recurrent somatic *DICER1* alterations, thereby adding evidence for a contributory role for this mutational inactivation also in sporadic thyroid tumors, including FTAs, FTCs and PTCs (18, 43, 44, 45). In adult patients, *RAS* gene family mutations predominantly characterize FTCs and FVPTCs, whereas *DICER1* mutations are relatively rare, found in less than 10% of sporadic FTAs and FTCs (31). Intriguingly, *DICER1* mutations are significantly more prevalent under specific conditions. In children, particularly those under 10 years old, a follicular-patterned thyroid tumor might be the initial manifestation of DICER1 syndrome, warranting genetic testing for a germline *DICER1* mutation. Overall, pediatric and adolescent patients with follicular-patterned thyroid tumors (FTAs/FTCs and FVPTCs) are enriched for somatic *DICER1* mutations (43, 45, 46). In addition, in young patients, the presence of coexistent TFND in association with a follicular-patterned tumor (especially FTA with papillary structures) should raise suspicion of *DICER1* aberrancies. In macrofollicular-predominant FTCs across all age groups, a majority of these neoplasms harbor *DICER1* mutations, sometimes coinciding with additional mutations (47, 48) (Fig. 5). Recent work has highlighted an additional possible genotype–phenotype correlation, where abortive/atrophic changes within the follicular-patterned nodules are strongly indicative of *DICER1* mutations (Fig. 5), which are often somatic in nature (37, 48, 49). Overall, *DICER1*-mutated follicular-patterned tumors are usually found in younger female patients, with distant metastases being rare or unreported.

**Figure 6:**
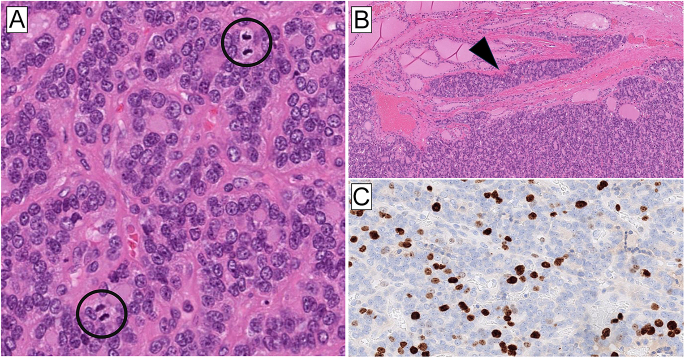
*DICER1*-mutated poorly differentiated thyroid carcinoma in an adolescent patient. (A) 50 mm thyroid tumor with biallelic *DICER1* mutations. The tumor fulfilled the Turin criteria for poorly differentiated thyroid carcinoma (PDTC), with a predominant solid to trabecular growth pattern and a lack of papillary thyroid carcinoma-related nuclear features. There were 10 mitotic figures per 2 mm^2^; two mitoses are marked by circles. (B) Invasive growth through the capsule was evident (arrowhead), and angioinvasion was present in several areas (not shown). (C) The Ki-67 proliferation index was 22%, which is in keeping with a diagnosis of PDTC.

Moreover, *DICER1* mutations have also been found in pediatric and young adult patients with PDTCs, while other classic driver mutations usually present in adult PDTCs are absent, suggesting a molecular distinction between pediatric and adult PDTCs (44, 50, 51) (Fig. 6). Although the available data are limited, emerging evidence indicates that these *DICER1*-associated PDTCs may exhibit a more aggressive clinical course, in contrast to most other *DICER1*-associated tumors, although there are exceptions to this rule (51). Finally, somatic *DICER1* mutations are usually reported in thyroblastoma, an embryonal high-grade triphasic thyroid neoplasm that is usually clinically aggressive (52).

It is important to note that biallelic *DICER1* somatic inactivation (through two separate mutations occurring in *trans*) is a common pattern across all *DICER1*-related thyroid neoplasms and does not automatically indicate germline involvement. However, considering the clinical benefit of identifying DICER1 syndromic patients, screening for germline mutations is recommended, particularly in young patients with a positive family history of thyroid tumors or lesions associated with DICER1 syndrome.

## *DGCR8* mutations in thyroid tumors

Although the initial observation of recurrent *DGCR8* germline mutations in patients with FMGS spurred the authors to investigate somatic DNA from a pool of sporadic thyroid tumors, no additional alterations were found in this cohort. However, shortly thereafter, Paulsson and colleagues identified *DGCR8* missense mutations (p.E518K) with concurrent LOH in highly aggressive FTCs, suggestive of biallelic inactivation (53). The authors speculated whether these mutations were progression events, as both tumors with this alteration already exhibited credible driver gene events (*PIK3CA* and *HRAS* mutations, respectively). Despite this, both *DGCR8*-mutated cases showed a distinct miRNA profile compared to *DGCR8* wild-type tumors, suggesting an effect on the global miRNA level. In addition, both cases with somatic *DGCR8* mutations exhibited evidence of distant metastases, indicating a potential correlation with poor prognosis. Moreover, the majority of FTCs in Paulsson’s cohort exhibited downregulation of *DGCR8* mRNA, further establishing *DGCR8* as a potential tumor suppressor gene in thyroid cancer. The findings of somatic *DGCR8* mutations and mRNA downregulation have since been reproduced, with the discovery of a single mutation in a case of poorly differentiated thyroid carcinoma and downregulation of *DGCR8* mRNA in a cohort of follicular-patterned thyroid neoplasia (54). To obtain reliable numbers regarding *DGCR8* mutational frequencies across different thyroid tumors, a combined effort from Italy and Sweden analyzed a total of 440 follicular-patterned thyroid lesions and found four additional cases (all malignant tumors) with somatic mutations, corresponding to 1% of the entire cohort (31). Mirroring the earlier observations by Paulsson and colleagues, *DGCR8* mutant cases displayed aggressive histology, and two out of four cases exhibited driver gene mutations in *RAS* family genes, providing further evidence for the theory that somatic *DGCR8* mutations may underlie the progression of thyroid cancer rather than initiate it. The reason for the development of TFND, when *DGCR8* is mutated in the germline setting, and the generation of aggressive thyroid carcinoma, when the same mutation is acquired at the somatic level, is not known, but it suggests that the timing of the mutational acquisition is crucial.

## The unique miRNA landscape of *DICER1*- and *DGCR8*-mutated thyroid carcinoma

The discovery of mutations in genes encoding members of the miRNA machinery prompted extensive investigations into thyroid tumors with this genetic abnormality to determine whether the miRNA pattern in these tumors was affected. Given that the majority of *DICER1* mutations are biallelic and inactivating in thyroid tumors and that *DGCR8* mutations usually occur with concurrent LOH, it was expected that these genetic alterations would impede the miRNA maturation process in the tumor cells. Indeed, a previous study of a pleuropulmonary blastoma (PPB) with biallelic *DICER1* mutations identified a unique reduction of 5p-derived mature miRNAs and an accumulation of 5p-derived pre-miRNAs, which are normally processed by a functional DICER1 protein (55). This study proposed that *DICER1* mutations led to an inability to cleave the 5p end of pre-miRNA hairpins.

Since then, gene-specific and global expression studies in thyroid tumors have demonstrated a range of disruptions in the miRNA pattern in cases with mutations in *DICER1* or *DGCR8*. For example, follicular thyroid tumors with *DICER1* mutations exhibit a unique miRNA pattern with a striking global loss of mature 5p miRNAs, similar to what was observed in PPB (18, 31). Moreover, *DGCR8*-mutated tumors also seem to demonstrate a unique miRNA profile compared to wild-type tumors of the same entity (53). In addition, *DICER1* and *DGCR8* mutants in thyroid tumors share a similar miRNA expression profile, distinguishing them from *DICER1*/*DGCR8* wild-type tumors. In one study, 427 differentially expressed miRNAs were found between wild-type and mutated cases, including miR-450a-1-3p, miR-548e-3p, miR-1246, miR-548n and miR-181a-3p as overexpressed in mutated cases, whereas five additional miRNAs (let-7i-5p, miR-135b-5p, miR135a-5p, miR3151-5p and miR-100-5p) were downregulated in mutated cases (31). Notably, many of these specific miRNAs have previously been reported to be dysregulated in thyroid cancer. However, the exact mechanism by which this dysregulation may cause a range of thyroid lesions, from benign and low-risk to high-grade carcinoma, is not yet known.

## Potential role of *DROSHA* and *XPO5* in thyroid carcinoma

The role of the other components of miRNA machinery, including *DROSHA*, *XPO5*, *TARBP2* and *AGO2*, in thyroid carcinogenesis remains unclear and largely unexplored. The mechanistic characterization of mutations in these genes and their potential oncogenic activities in thyroid cancer are not well understood, highlighting a significant gap in our knowledge of their contribution to thyroid tumorigenesis.

Frequent heterozygous somatic mutations in *DROSHA* have been identified in rare pediatric forms of Wilms tumors. Functional analyses revealed that these mutations affect the synthesis of miRNAs from both pre-miRNA arms 3p and 5p, suggesting a potential oncogenic role in these tumors (56). In thyroid cancer, Paulsson and coworkers reported a point mutation in the *DROSHA* gene, specifically investigating a rare and unique case of synchronous FTC/PDTC/ATC from a single patient. This study provided evidence of a missense p.R277C mutation, alongside other progression events involving *TP53*, *TERT* and *APC* genes, found only in the PDTC component of the tumor (57). Alterations in *DROSHA* have also been identified in benign FTAs and encapsulated FVPTCs by Poma and collaborators. Subsequent analysis of the paired normal tissue revealed the germline origin of these alterations, and the variants discovered were synonymous (p.S981S and p.Y1199Y). Notably, two out of four tumors with germline *DROSHA* variants also harbored *RAS* driver mutations (18). These findings suggest a potential, albeit unclear, role of *DROSHA* alterations in thyroid tumorigenesis and their contribution to the dedifferentiation process. Further exploration into the role of this gene in thyroid cancer development is required.

Following the microprocessor activities in the nucleus, pre-miRNAs are exported to the cytoplasm by the XPO5 protein. Recent studies have reported specific indel hotspots in the *XPO5* gene in cancers with a high rate of microsatellite instability, such as colon, gastric and endometrial tumors (58). However, these alterations have not been further investigated in other studies. Nevertheless, very little is known about the potential role of *XPO5* alteration in thyroid tumors. A putative *XPO5::CHST9* fusion in ATC has also been described; however, the true functional consequences of this fusion in the development of ATC remain unknown (59).

Mutations in the *TARBP2* gene have been associated with tumorigenesis in certain cancers. For example, *TARBP2* mutations have been identified in upper urinary tract urothelial carcinomas, correlating with microsatellite instability. However, mutations in *AGO2* are not as frequently reported as those in other components of the miRNA machinery.

## Implication for clinical management

Patients with DICER1 syndrome are at an elevated risk of various neoplasms, including DTC, pleuropulmonary blastoma and other rare tumors. Clinical management emphasizes early detection and monitoring, particularly during childhood and adolescence, when the risk of certain tumors, such as pleuropulmonary blastoma, is highest. Some guidelines recommend that patients with DICER1 syndrome undergo annual clinical examinations from birth through age 20, with thyroid ultrasounds every three years from ages 8 to 40 (60). Specific screening guidelines for *DGCR8* mutation carriers are not well established due to the rarity of this mutation. However, given its association with aggressive thyroid cancers, frequent thyroid imaging may be prudent. Future studies will likely clarify optimal screening protocols for these patients.

## Discussion and future aspects

The identification of the miRNA machinery as a key regulator of gene expression has enhanced our understanding of how cells control the expression of specific gene signatures, which in turn impacts metabolism, growth and differentiation. The miRNA apparatus has been shown to be dysregulated in many different tumor types, and significant efforts have been made to identify which miRNAs are aberrantly expressed in tumors and how these contribute to tumorigenesis. Furthermore, the discovery of specific tumor syndromes linked to constitutional mutations in genes encoding members of this miRNA machinery, such as *DICER1* and *DGCR8*, has revealed *bona fide* tumor suppressor functions for these genes. Since both DICER1 and FMGS syndromes are associated with thyroid disease, it is logical for researchers to also investigate the expression and sequencing of the responsible genes in sporadic thyroid tumors. Indeed, somatic inactivation of *DICER1* appears to occur in a wide range of thyroid lesions, from TFND and FTAs to poorly differentiated thyroid cancer. Some patients have an underlying constitutional mutation and acquire a second somatic mutation on the other allele, while others develop biallelic inactivation through somatic mutations alone. The reason why some patients with germline mutations develop benign conditions while others develop aggressive thyroid cancer is not fully understood. In addition, the significance of biallelic *DICER1* mutations in both benign and highly malignant conditions remains unclear. Interestingly, *DICER1*-mutated lesions are rarely accompanied by other driver mutations, which may indicate that a subgroup lacks a primary driver for tumor development. However, there are likely other unknown genetic events, in addition to *DICER1* mutations, that influence the development of benign and malignant diseases.

In terms of tumor development, the unique miRNA landscape of *DICER*1- and *DGCR8*-mutated thyroid carcinoma, especially the shift from 5p to 3p miRNA expression, could provide important clues for future investigations into how the inactivation of miRNA regulators may drive or promote thyroid cancer development and/or dedifferentiation. Understanding these mechanisms could pave the way for novel therapeutic strategies aimed at manipulating miRNA patterns to combat thyroid cancer more effectively.

From a clinical perspective, *DICER1* mutations are seen across the entire spectrum of thyroid lesions, and it is crucial to avoid missing a syndromic carrier in routine clinical settings. Clues to triaging cases for genetic testing may include a young age of onset, female sex, various histotypes (such as TFND, follicular adenoma with papillary structures, macrofollicular thyroid tumors and PDTCs) and specific histological indicators (such as atrophic changes and papillary infoldings). While the prognosis for *DICER1*-mutated well-differentiated thyroid carcinoma is usually good, it is much poorer for PDTCs in pediatric patients. *DGCR8* mutations are typically found in high-grade thyroid tumors and may represent a progression event similar to *TERT* promoter mutations rather than a primary driver event, as these mutations are often accompanied by *bona fide* driver mutations in thyroid-related genes.

Detecting a *DICER1* mutation preoperatively could be valuable, as this mutation may be constitutional and suggest hereditary disease. However, because somatic mutations in *DICER1* are found in benign and low-grade malignant lesions and in pediatric PDTCs, their clinical utility in terms of diagnostic potential remains limited. Conversely, the presence of somatic *DGCR8* mutations, although rare, appears concentrated in highly aggressive thyroid carcinomas, suggesting potential preoperative value if routine molecular screening was implemented. While further data are needed, finding a *DGCR8* mutation in a thyroid aspirate may support a diagnosis of malignancy. Currently, no evidence indicates that *DGCR8*- or *DICER1*-mutated thyroid tumors exhibit specific features easily recognizable during the preoperative cytological assessment, although this will be a subject of intensified research in the near future.

From a broader healthcare perspective, understanding the roles of *DICER1* and *DGCR8* mutations in thyroid tumors is vital, as it highlights the importance of genetic testing and personalized medicine in managing thyroid cancer. Early identification of these mutations can guide treatment decisions and improve patient outcomes, particularly in pediatric and high-risk populations. This knowledge also underscores the potential for developing targeted therapies that could transform the clinical management of thyroid cancer, moving toward more precise and effective treatment options.

## Declaration of interest

The authors declare that there is no conflict of interest that could be perceived as prejudicing the impartiality of the work.

## Funding

This work did not receive any specific grant from any funding agency in the public, commercial or not-for-profit sector.

## Author contribution statement

Both authors (VC and CCJ) conceived the study and wrote the paper.

## References

[1] Ha M & Kim VN Regulation of microRNA biogenesis. Nat Rev Mol Cell Biol 2014 15 509–524. (https://www.nature.com/articles/nrm3838) 25027649 10.1038/nrm3838

[2] Lee RC, Feinbaum RL & Ambros V The *C. elegans* heterochronic gene lin-4 encodes small RNAs with antisense complementarity to lin-14. Cell 1993 75 843–854. (10.1016/0092-8674(93)90529-y) 8252621

[3] Ambros V microRNAs: tiny regulators with great potential. Cell 2001 107 823–826. (10.1016/s0092-8674(01)00616-x) 11779458

[4] Reinhart BJ, Slack FJ, Basson M, et al. The 21-nucleotide let-7 RNA regulates developmental timing in *Caenorhabditis elegans*. Nature 2000 403 901–906. (https://www.nature.com/articles/35002607) 10706289 10.1038/35002607

[5] Ludwig N, Leidinger P, Becker K, et al. Distribution of miRNA expression across human tissues. Nucleic Acids Res 2016 44 3865–3877. (https://academic.oup.com/nar/article-lookup/doi/10.1093/nar/gkw116) 26921406 10.1093/nar/gkw116 PMC4856985

[6] Calin GA, Sevignani C, Dumitru CD, et al. Human microRNA genes are frequently located at fragile sites and genomic regions involved in cancers. Proc Natl Acad Sci U S A 2004 101 2999–3004. (https://pnas.org/doi/full/10.1073/pnas.0307323101) 14973191 10.1073/pnas.0307323101 PMC365734

[7] Di Leva G, Garofalo M & Croce CM MicroRNAs in cancer. Annu Rev Pathol 2014 9 287–314. (https://www.annualreviews.org/doi/10.1146/annurev-pathol-012513-104715) 24079833 10.1146/annurev-pathol-012513-104715 PMC4009396

[8] Guo Z, Maki M, Ding R, et al. Genome-wide survey of tissue-specific microRNA and transcription factor regulatory networks in 12 tissues. Sci Rep 2014 4 5150. (https://www.nature.com/articles/srep05150) 24889152 10.1038/srep05150 PMC5381490

[9] Peng Y & Croce CM The role of MicroRNAs in human cancer. Signal Transduct Target Ther 2016 1 15004. (http://www.nature.com/articles/sigtrans20154) 29263891 10.1038/sigtrans.2015.4 PMC5661652

[10] Cimmino A, Calin GA, Fabbri M, et al. miR-15 and miR-16 induce apoptosis by targeting BCL2. Proc Natl Acad Sci U S A 2005 102 13944–13949. (10.1073/pnas.0506654102) 16166262 PMC1236577

[11] Svoronos AA, Engelman DM & Slack FJ OncomiR or tumor suppressor? The duplicity of MicroRNAs in cancer. Cancer Res 2016 76 3666–3670. (https://aacrjournals.org/cancerres/article/76/13/3666/608115/OncomiR-or-Tumor-Suppressor-The-Duplicity-of) 27325641 10.1158/0008-5472.CAN-16-0359 PMC4930690

[12] O’Brien J, Hayder H, Zayed Y, et al. Overview of MicroRNA biogenesis, mechanisms of actions, and circulation. Front Endocrinol 2018 9 402. (10.3389/fendo.2018.00402) PMC6085463 30123182

[13] Stavast CJ & Erkeland SJ The non-canonical aspects of MicroRNAs: many roads to gene regulation. Cells 2019 8 E1465. (10.3390/cells8111465) PMC6912820 31752361

[14] Ruby JG, Jan CH & Bartel DP Intronic microRNA precursors that bypass Drosha processing Nature 2007 448 83–86. (http://www.nature.com/articles/nature05983) 17589500 10.1038/nature05983 PMC2475599

[15] Cheloufi S, Dos Santos CO, Chong MMW, et al. A dicer-independent miRNA biogenesis pathway that requires ago catalysis. Nature 2010 465 584–589. (http://www.nature.com/articles/nature09092) 20424607 10.1038/nature09092 PMC2995450

[16] Baloch ZW, Asa SL, Barletta JA, et al. Overview of the 2022 WHO classification of thyroid neoplasms. Endocr Pathol 2022 33 27–63. (https://link.springer.com/10.1007/s12022-022-09707-3) 35288841 10.1007/s12022-022-09707-3

[17] Condello V, Torregrossa L, Sartori C, et al. mRNA and miRNA expression profiling of follicular variant of papillary thyroid carcinoma with and without distant metastases. Mol Cell Endocrinol 2019 479 93–102. (10.1016/j.mce.2018.09.005) 30261209

[18] Poma AM, Condello V, Denaro M, et al. DICER1 somatic mutations strongly impair miRNA processing even in benign thyroid lesions. Oncotarget 2019 10 1785–1797. (10.18632/oncotarget.26639) 30956758 PMC6442996

[19] He H, Jazdzewski K, Li W, et al. The role of microRNA genes in papillary thyroid carcinoma. Proc Natl Acad Sci U S A 2005 102 19075–19080. (10.1073/pnas.0509603102) 16365291 PMC1323209

[20] Weber F, Teresi RE, Broelsch CE, et al. A limited set of human MicroRNA is deregulated in follicular thyroid carcinoma. J Clin Endocrinol Metab 2006 91 3584–3591. (https://academic.oup.com/jcem/article/91/9/3584/2656761) 16822819 10.1210/jc.2006-0693

[21] Visone R, Pallante P, Vecchione A, et al. Specific microRNAs are downregulated in human thyroid anaplastic carcinomas. Oncogene 2007 26 7590–7595. (https://www.nature.com/articles/1210564) 17563749 10.1038/sj.onc.1210564

[22] Nikiforova MN, Tseng GC, Steward D, et al. MicroRNA expression profiling of thyroid tumors: biological significance and diagnostic utility. J Clin Endocrinol Metab 2008 93 1600–1608. (10.1210/jc.2007-2696) 18270258 PMC2386678

[23] Borrelli N, Denaro M, Ugolini C, et al. miRNA expression profiling of ‘noninvasive follicular thyroid neoplasms with papillary-like nuclear features’ compared with adenomas and infiltrative follicular variants of papillary thyroid carcinomas. Mod Pathol 2017 30 39–51. (10.1038/modpathol.2016.157) 27586203

[24] Denaro M, Ugolini C, Poma AM, et al. Differences in miRNA expression profiles between wild-type and mutated NIFTPs. Endocr Relat Cancer 2017 24 543–553. (10.1530/erc-17-0167) 28830935

[25] Misiak D, Bauer M, Lange J, et al. MiRNA deregulation distinguishes anaplastic thyroid carcinoma (ATC) and supports upregulation of oncogene expression. Cancers 2021 13 5913. (https://www.mdpi.com/2072-6694/13/23/5913) 34885022 10.3390/cancers13235913 PMC8657272

[26] Agrawal N, Akbani R, Aksoy B, et al. Integrated genomic characterization of papillary thyroid carcinoma. Cell 2014 159 676–690. (10.1016/j.cell.2014.09.050) 25417114 PMC4243044

[27] Pennelli G, Galuppini F, Barollo S, et al. The PDCD4/miR-21 pathway in medullary thyroid carcinoma. Hum Pathol 2015 46 50–57. (https://linkinghub.elsevier.com/retrieve/pii/S0046817714003724) 25316501 10.1016/j.humpath.2014.09.006

[28] Hill DA, Ivanovich J, Priest JR, et al. DICER1 mutations in familial pleuropulmonary blastoma. Science 2009 325 965. (10.1126/science.1174334) 19556464 PMC3098036

[29] Kim J, Schultz KAP, Hill DA, et al. The prevalence of germline DICER1 pathogenic variation in cancer populations. Mol Genet Genomic Med 2019 7 e555. (10.1002/mgg3.555) 30672147 PMC6418698

[30] Anglesio MS, Wang Y, Yang W, et al. Cancer‐associated somatic *DICER1* hotspot mutations cause defective miRNA processing and reverse‐strand expression bias to predominantly mature 3p strands through loss of 5p strand cleavage. J Pathol 2013 229 400–409. (10.1002/path.4135) 23132766

[31] Condello V, Poma AM, Macerola E, et al. Prevalence, molecular landscape, and clinical impact of DICER1 and DGCR8 mutated follicular-patterned thyroid nodules. J Clin Endocrinol Metab 2024 109 1733–1744. (10.1210/clinem/dgae034) 38252873 PMC11180504

[32] Seki M, Yoshida K, Shiraishi Y, et al. Biallelic DICER1 mutations in sporadic pleuropulmonary blastoma. Cancer Res 2014 74 2742–2749. (10.1158/0008-5472.can-13-2470) 24675358

[33] Wu MK, Sabbaghian N, Xu B, et al. Biallelic *DICER1* mutations occur in Wilms tumours. J Pathol 2013 230 154–164. (10.1002/path.4196) 23620094

[34] de Kock L, Rivera B, Revil T, et al. Sequencing of DICER1 in sarcomas identifies biallelic somatic DICER1 mutations in an adult-onset embryonal rhabdomyosarcoma. Br J Cancer 2017 116 1621–1626. (10.1038/bjc.2017.147) 28524158 PMC5518865

[35] Wu MK, Vujanic GM, Fahiminiya S, et al. Anaplastic sarcomas of the kidney are characterized by DICER1 mutations. Mod Pathol 2018 31 169–178. (10.1038/modpathol.2017.100) 28862265

[36] de Kock L, Sabbaghian N, Soglio DBD, et al. Exploring the association between DICER1 mutations and differentiated thyroid carcinoma. J Clin Endocrinol Metab 2014 99 E1072–E1077. (10.1210/jc.2013-4206) 24617712

[37] Condello V, Roberts JW, Stenman A, et al. Atrophic changes in thyroid tumors are strong indicators of underlying DICER1 mutations: a bi-institutional genotype-phenotype correlation study. Virchows Arch 2024 485 105–114. (10.1007/s00428-024-03802-y) 38637342 PMC11271315

[38] Rivera B, Nadaf J, Fahiminiya S, et al. DGCR8 microprocessor defect characterizes familial multinodular goiter with schwannomatosis. J Clin Invest 2020 130 1479–1490. (10.1172/jci130206) 31805011 PMC7269565

[39] Altaraihi M, Hansen TVO, Santoni-Rugiu E, et al. Prevalence of pathogenic germline DICER1 variants in young individuals thyroidectomised due to goitre – a national Danish cohort. Front Endocrinol 2021 12 727970. (10.3389/fendo.2021.727970) PMC8451242 34552563

[40] de Kock L, Bah I, Revil T, et al. Deep sequencing reveals spatially distributed distinct hot spot mutations in DICER1-related multinodular goiter. J Clin Endocrinol Metab 2016 101 3637–3645. (10.1210/jc.2016-1328) 27459524

[41] Namba H, Rubin SA & Fagin JA Point mutations of ras oncogenes are an early event in thyroid tumorigenesis. Mol Endocrinol 1990 4 1474–1479. (10.1210/mend-4-10-1474) 2283998

[42] Krohn K, Führer D, Bayer Y, et al. Molecular pathogenesis of euthyroid and toxic multinodular goiter. Endocr Rev 2005 26 504–524. (10.1210/er.2004-0005) 15615818

[43] Wasserman JD, Sabbaghian N, Fahiminiya S, et al. DICER1 mutations are frequent in adolescent-onset papillary thyroid carcinoma. J Clin Endocrinol Metab 2018 103 2009–2015. (10.1210/jc.2017-02698) 29474644

[44] Chernock RD, Rivera B, Borrelli N, et al. Poorly differentiated thyroid carcinoma of childhood and adolescence: a distinct entity characterized by DICER1 mutations. Mod Pathol 2020 33 1264–1274. (10.1038/s41379-020-0458-7) 31937902 PMC7329587

[45] Lee YA, Im SW, Jung KC, et al. Predominant DICER1 pathogenic variants in pediatric follicular thyroid carcinomas. Thyroid 2020 30 1120–1131. (10.1089/thy.2019.0233) 32228164

[46] Onder S, Mete O, Yilmaz I, et al. DICER1 mutations occur in more than one-third of follicular-patterned pediatric papillary thyroid carcinomas and correlate with a low-risk disease and female gender predilection. Endocr Pathol 2022 33 437–445. (10.1007/s12022-022-09736-y) 36251117

[47] Hellgren LS, Hysek M, Jatta K, et al. Macrofollicular variant of follicular thyroid carcinoma (MV-FTC) with a somatic DICER1 gene mutation: case report and review of the literature. Head Neck Pathol 2021 15 668–675. (10.1007/s12105-020-01208-1) 32712880 PMC8134796

[48] Juhlin CC, Stenman A & Zedenius J Macrofollicular variant follicular thyroid tumors are DICER1 mutated and exhibit distinct histological features. Histopathology 2021 79 661–666. (10.1111/his.14416) 34008223

[49] Jung CK, Liu Z, Hirokawa M, et al. Histological clues of *DICER1* mutations in thyroid nodules. Virchows Arch 2024 485 755–757. (https://link.springer.com/10.1007/s00428-024-03915-4) 39231821 10.1007/s00428-024-03915-4

[50] Ver Berne J, Van den Bruel A, Vermeire S, et al. DICER1 mutations define the landscape of poorly differentiated thyroid carcinoma in children and young adults: case report and literature review. Am J Surg Pathol 2024 48 1277–1283. (10.1097/pas.0000000000002265) 38912716

[51] Yegen G, Altay AY, Yılmaz İ, et al. DICER1 mutations do not always indicate dismal prognosis in pediatric poorly differentiated thyroid carcinomas. Endocr Pathol 2023 34 279–286. (10.1007/s12022-023-09780-2) 37574466

[52] Erickson LA, Rivera M, Guo R, et al. Thyroblastoma-a primitive multilineage thyroid neoplasm with somatic DICER1 alteration. Endocr Pathol 2023 34 159–160. (10.1007/s12022-023-09750-8) 36790721

[53] Paulsson JO, Rafati N, DiLorenzo S, et al. Whole-genome sequencing of follicular thyroid carcinomas reveal recurrent mutations in MicroRNA processing subunit DGCR8. J Clin Endocrinol Metab 2021 106 3265–3282. (10.1210/clinem/dgab471) 34171097 PMC8530729

[54] Rodrigues L, Canberk S, Macedo S, et al. DGCR8 microprocessor subunit mutation and expression deregulation in thyroid lesions. Int J Mol Sci 2022 23 14812. (10.3390/ijms232314812) 36499151 PMC9740158

[55] Pugh TJ, Yu W, Yang J, et al. Exome sequencing of pleuropulmonary blastoma reveals frequent biallelic loss of TP53 and two hits in DICER1 resulting in retention of 5p-derived miRNA hairpin loop sequences. Oncogene 2014 33 5295–5302. (10.1038/onc.2014.150) 24909177 PMC4224628

[56] Torrezan GT, Ferreira EN, Nakahata AM, et al. Recurrent somatic mutation in DROSHA induces microRNA profile changes in Wilms tumour. Nat Commun 2014 5 4039. (https://www.nature.com/articles/ncomms5039) 24909261 10.1038/ncomms5039 PMC4062040

[57] Paulsson JO, Backman S, Wang N, et al. Whole‐genome sequencing of synchronous thyroid carcinomas identifies aberrant DNA repair in thyroid cancer dedifferentiation. J Pathol 2020 250 183–194. (https://pathsocjournals.onlinelibrary.wiley.com/doi/10.1002/path.5359) 31621921 10.1002/path.5359

[58] Melo SA, Moutinho C, Ropero S, et al. A genetic defect in exportin-5 traps precursor MicroRNAs in the nucleus of cancer cells. Cancer Cell 2010 18 303–315. (https://linkinghub.elsevier.com/retrieve/pii/S1535610810003442) 20951941 10.1016/j.ccr.2010.09.007

[59] Stenman A, Yang M, Paulsson JO, et al. Pan-genomic sequencing reveals actionable *CDKN2A/2B* deletions and kataegis in anaplastic thyroid carcinoma. Cancers 2021 13 6340. (10.3390/cancers13246340) 34944959 PMC8699293

[60] Bakhuizen JJ, Hanson H, van der Tuin K, et al. Surveillance recommendations for DICER1 pathogenic variant carriers: a report from the SIOPE host genome working group and CanGene-CanVar clinical guideline working group. Fam Cancer 2021 20 337–348. (10.1007/s10689-021-00264-y) 34170462 PMC8484187

